# PsychoBehavioroimmunology: Connecting the Behavioral Immune System to Its Physiological Foundations

**DOI:** 10.3389/fpsyg.2019.00200

**Published:** 2019-02-07

**Authors:** Damian R. Murray, Marjorie L. Prokosch, Zachary Airington

**Affiliations:** Department of Psychology, Tulane University, New Orleans, LA, United States

**Keywords:** behavioral immune system, psychoneuroimmunology, health, immune system regulation, social cognition

## Abstract

Although infectious disease has posed a significant and persistent threat to human survival and welfare throughout history, only recently have the psychological and behavioral implications of disease threat become a topic of research within the behavioral sciences. This growing body of work has revealed a suite of affective and cognitive processes that motivate the avoidance of disease-causing objects and situations—a cascade of processes loosely conceptualized as a “behavioral immune system (BIS).” Recent BIS research has linked disease threat to a surprisingly broad set of psychological and behavioral phenomena. However, research examining how the BIS is nested within our broader physiology is only beginning to emerge. Here, we review research that has begun to elucidate the physiological foundations of the BIS—at the levels of sensory modalities, cells, and genes. We also discuss the future of this work.

## Psychobehavioroimmunology: Connecting the Behavioral Immune System to Its Physiological Foundations

The threat posed by infectious disease throughout human evolution has likely caused more deaths than all other causes of mortality combined (e.g., Inhorn and Brown, 1990). This threat is not unique to humans; many biologists characterize the evolution of all animal species as having been driven substantially by a billion-year evolutionary arms race between parasites and their hosts (Zuk, 1992, 2007; Knoll and Carroll, 1999; Zimmer, 2001), with viruses alone estimated to be responsible for one third of mammalian genetic adaptations (Enard et al., 2016). One product of this arms race in humans (and other vertebrates) is the immune system, which is comprised of an astonishingly complex set of mechanisms which reactively vanquish infections when they occur (a system that has the capacity to generate billions of unique antibodies; Fanning et al., 1996; Janeway, 2001). Given the fitness costs of immune system activation, however, humans also proactively respond to disease-connoting cues with a cascade of affective and cognitive responses which, in turn, motivate behavior that minimizes the probability of infection—a psychobehavioral agglomerate now popularly referred to as the *behavioral* immune system (BIS) (see Schaller and Park, 2011; Murray and Schaller, 2016; Ackerman et al., 2018a). But until recently, little work investigated the ways in which the “behavioral” and physiological immune systems interact. Here, we provide an overview of burgeoning research linking the BIS to its physiological foundations.

### Recent Behavioral Immune System Research

Behavioral immune system research suggests that the social and behavioral implications of perceived disease threat range from basic perceptual processes (e.g., facial perception), to judgment and decision-making processes, to culture-wide norms and social systems (for reviews see Murray and Schaller, 2014, 2016, 2017). Each of these related lines of research are theoretically underpinned by a distinct conceptual hypothesis which considers the cost/benefit ratio of a given trait or behavior, and how this ratio is variable dependent upon the threat of disease (or perceived threat of disease) within one’s environment. For example, whereas gregarious social behavior, risk taking, and promiscuous sexuality all have distinct, unique benefits, they are similar in that high levels of each are associated with disease-specific costs. This costs/benefit logic implies the hypotheses that greater infection threat will be associated with lower gregariousness, lower risk tolerance, and less promiscuous sexuality. Several studies now provide evidence for these hypotheses, using both trait measures of perceived disease threat and laboratory manipulations of disease threat (Mortensen et al., 2010; Murray et al., 2013; Sparks et al., 2018; Prokosch et al., in press), as well as ecological variation in actual disease threat (Schaller and Murray, 2008; Murray and Schaller, 2010; Van Leeuwen et al., 2012; Murray, 2014a,b). Similar implicit cost/benefit logic has also been employed to experimentally link disease threat to outgroup stereotyping and prejudice (Faulkner et al., 2004; Park et al., 2007; Huang et al., 2011), conformity (Murray and Schaller, 2012; Wu and Chang, 2012; Murray et al., 2019c), anticipated future sexual behavior (Hill et al., 2015), and self-image concern (Ackerman et al., 2018b).

### Integrating Behavioral and Physiological Immunity

Until relatively recently, BIS research proceeded without much theoretical regard to what the physiological underpinnings of this “system” might be. This is unsurprising given that this research was conducted predominantly in social psychology labs. Therefore, the majority of this work ignored the foundational question of how psychological and behavioral disease avoidance strategies are intrinsically embedded within the immune system proper. In doing so, this work paid little homage to the vast literature comprising the field of psychoneuroimmunology (PNI), which for decades has studied the interactions between the immune system, the brain, and behavior (e.g., see Clark and Fessler, 2014).

This isolated state of affairs is shifting. A growing body of work in the psychological sciences is beginning to elucidate how the BIS is influenced by—and influences—the physiological immune system. We believe that is work is both conceptually related to, but currently distinct from, that which characterizes PNI research for at least two reasons. The first reason is a matter of scale: whereas PNI research frequently concerns questions regarding mechanistic pathways connecting the immune system to the brain (e.g., the immune-brain loop or cell-signaling networks, see Daruna, 2012), physiological BIS research to date is primarily concerned with the relationships between immune processes and overt social cognition and behavior. A second reason is that whereas PNI is more primarily focused on *reactive* psychological responses to already-existing infection (such as sickness behavior, e.g., see Dantzer and Kelley, 2007) or the dysregulation of otherwise adaptive systems (such as cytokine-induced depression, e.g., see Loftis et al., 2010), research investigating the physiological correlates of the BIS is more primarily concerned with how *proactive* (and sometimes ostensibly unrelated) behaviors that minimize infection risk are associated with the immune system. Here, we provide an overview of this emerging subfield.

## Physiological Foundations of the Behavioral Immune System

Recent work expanding our understanding of the physiological foundations of the BIS can be parsed into three levels of analysis: Sensory, cellular, and genetic. We review each of these below.

### Sensory Components

Early and current work investigating the implications of experimentally “activating” the BIS has predominantly used visual cues of pathogenic risk. However, recent work is elucidating the importance of other sensory modalities in disease detection and its coordinated BIS responses as well.

Some work suggests that olfactory cues of disease not only elicit disgust but also predict prophylactic behaviors. In non-human animal populations, body odorants from conspecifics can convey infection status (mice; Kavaliers et al., 2005; bullfrog tadpoles; Kiesecker et al., 1999). In humans, body odors may convey similar information. Body odors from sick targets are rated less desirable and likeable (Regenbogen et al., 2017), and are also evaluated as unhealthier, more intense, and less pleasant (Olsson et al., 2014). Disgust ratings of body odors are also dependent upon the odor source, with odors of closer family members rated as less disgusting than strangers’ body odors (Stevenson and Repacholi, 2005; Case et al., 2006). The strong association between body odor and disgust has even recently inspired a unique psychometric measure, the Body Odor Disgust Scale (BODS; Liuzza et al., 2017).

Interestingly, body odorants may also communicate someone’s state disgust. Individuals who smelled body odors from participants primed with disgust had disgust-related reactions (e.g., nose wrinkling, reduced visual scanning; de Groot et al., 2012). Other work suggests that, in addition to activation of the neural structures associated with disgust (Wicker et al., 2003), experimentally inducing aversive odors also leads to patently prophylactic cognitions (greater intentions to use condoms; Tybur et al., 2011) and even less-directly prophylactic cognitions (condemnation of moral violations; Schnall et al., 2008). These results speak to the associations between olfactory detection, disease avoidance, and specific affective and behavioral reactions.

Emerging work is also beginning to link olfactory *acuity* to disgust and associated BIS responses. Given that olfactory cues are often subtle and their meanings ambiguous, a greater ability to detect such cues may be associated with more affective vigilance toward disease cues more generally. Recent work suggests that higher olfactory acuity may be associated with higher avoidance motivation (Fay and Bovier, 2018). Similarly, Murray et al. (unpublished) found that greater olfactory acuity—operationalized as a greater ability to detect, discriminate between, and identify odors—predicted greater sexual disgust and more restricted sociosexuality. However, olfactory acuity was not meaningfully associated with Perceived Vulnerability to Disease (Duncan et al., 2009). Such investigations remain a work in progress.

While work examining olfactory and visual processes in disease detection abounds in comparison with other sensory modalities, there is emerging evidence that the disease avoidance toolkit employs our full complement of senses. Gustatory stimuli, particularly perishable food sources, evoke feelings of disgust and avoidance when paired with disease primes (Tybur et al., 2016). Higher disgust sensitivity also positively predicts aversion to novel or foreign foods (Al-Shawaf et al., 2015). Auditory and tactile senses are also impacted by behavioral immune responses: Visual disease primes increase tactile sensitivity, and lead to perceptions of individuals with foreign accents as more distant, especially for participants scoring higher in disgust sensitivity (Reid et al., 2012; Hunt et al., 2017). Human-specific disgust sensitivity also predicts people’s preferred amount of personal space (Park, 2015).

As multi-sensory work grows, increasingly creative examinations of sensory phenomena and their affective reactions are also emerging. For example, Blake et al. (2017) investigated the “heebie-jeebies” as a disease-avoidance response that is distinct from disgust and fear. Taken together, a multi-sensory approach will be increasingly necessary in studying BIS processes.

### Cellular Correlates

Broadly, the immune system proper is comprised of dozens of biological components and cellular-level processes of varying specificity that act to detect and neutralize invading pathogens. New research programs are now elucidating how the BIS interacts with these cellular-level processes. Early attempts to study the interplay between the BIS and the body examined how acute environmental disease cues influence immune function. Experimental studies reveal that exposure to visual cues of contagion—in addition to increasing disgust and prejudice responses—upregulate oral and blood immune inflammatory biomarkers (Schaller et al., 2010; Stevenson et al., 2011, 2012, 2015; Makhanova et al., unpublished). For example, men who viewed pathogenic pictures (relative to control images) reported greater disgust, had higher body temperature, and had greater salivary concentrations of TNF-alpha and albumin (Stevenson et al., 2012). Similarly, participants’ blood plated *in-vitro* released more IL-6 in response to mitogen stimulation (by LPS) after they viewed pathogenic pictures (Schaller et al., 2010). Such results suggest that visual components of the BIS may proactively induce immunological responses when the threat of infection appears imminent.

However, correlational studies suggest that in the absence of immediate pathogen threat, the relationship between the BIS and physiological immune system may not always be additive. For example, stimulated release of proinflammatory cytokines (IL-6, IL-1β, TNF-alpha) from blood collected from healthy subjects (absent experimental primes) were not related to self-reported germ aversion (Gassen et al., 2018). However, these same participants’ spontaneously released (IL-6, IL-1β, TNF-a) and *in-vivo* proinflammatory cytokine (IL-6) levels were *negatively* related to germ aversion and perceived longevity. Taken together, these results suggest that in addition to short-term pathogen management, the BIS may also function to promote long-term health by lowering levels of basal inflammation and cellular oxidation. Relatedly, while IL-6 has traditionally been highlighted for its proinflammatory (i.e., defense) qualities, it also serves downstream anti-inflammatory tissue maintenance functions (Del Giudice and Gangestad, 2018). Future work will unpack the similar or dissimilar functions that defense versus maintenance IL-6 may serve in cueing behavioral defense strategies.

New research also highlights personal control over pathogen exposure as a potential key factor predicting investment in behavioral (e.g., avoidance) versus physiological (e.g., tolerance) immunity. Participants who reported lesser ability to avoid pathogenic threats in their day-to-day lives had a higher white blood cell count and, in turn, slower avoidance of pathogenic stimuli on an approach-avoidance task (Bradshaw et al., unpublished). In an experimental follow-up, ingesting an inflammation-suppressing aspirin led participants to report greater avoidance motivation toward pathogenic stimuli. Non-human research conceptually mirrors these results—social insect species that more effectively behaviorally manage pathogens at the colony level (e.g., by resin-lining their nests) display lower physiological immunity at the individual level (López-Uribe et al., 2016).

Other research has examined how physiological needs impact behavioral immune activity. One study found that men who have a profile indicative of strong physiological immunity (i.e., high testosterone and low cortisol) have weaker behavioral immune responses, as indexed by facial preferences (Kandrik et al., 2017). Conversely, recently ill and frequently ill people show greater BIS activation (e.g., greater disgust, avoidance of others) than healthier peers (Stevenson et al., 2009; Miller and Maner, 2011). Similarly, women experience elevated disgust and ethnocentrism during early pregnancy, when immunosuppression is greatest (Fessler et al., 2005; Navarrete et al., 2007). Preliminary hypotheses posited that progesterone-induced immunosuppression prompts compensatory prophylactic activity (Fleischman and Fessler, 2011), but replication attempts have not found a robust link between progesterone and pathogen-avoidant behaviors. Thus, the roles that progesterone and other pregnancy-related hormones play in evoking BIS responses remain unclear (Jones et al., 2018).

### Genetic Correlates

Although the majority of between-person variation in immune function appears to be due to non-heritable factors (see Brodin and Davis, 2017), genetic variation matters for disease vulnerability as well. Other BIS-motivated research is beginning to uncover how genes influence—and are influenced by—overt disease related behaviors and situations. One line of research examines individual differences in the genetic bases of immunocompetence and their relation to individual differences in behavioral tendencies associated with the BIS. One study focused on the IFNG +874 gene, one allele of which is associated with greater susceptibility to infectious diseases such as malaria, tuberculosis, and leprosy. Results revealed that individuals who possessed the disease-risk allele reported generally lower levels of extraversion and higher levels of harm avoidance (MacMurray et al., 2014). Another study focused on a different genetic polymorphism—the ACP1 gene—that also has a specific allele (the C allele) which is associated with poorer immunological function. Individuals who possessed the C allele reported lower levels of both extraversion and openness to experience (Napolioni et al., 2014).

**FIGURE 1:**
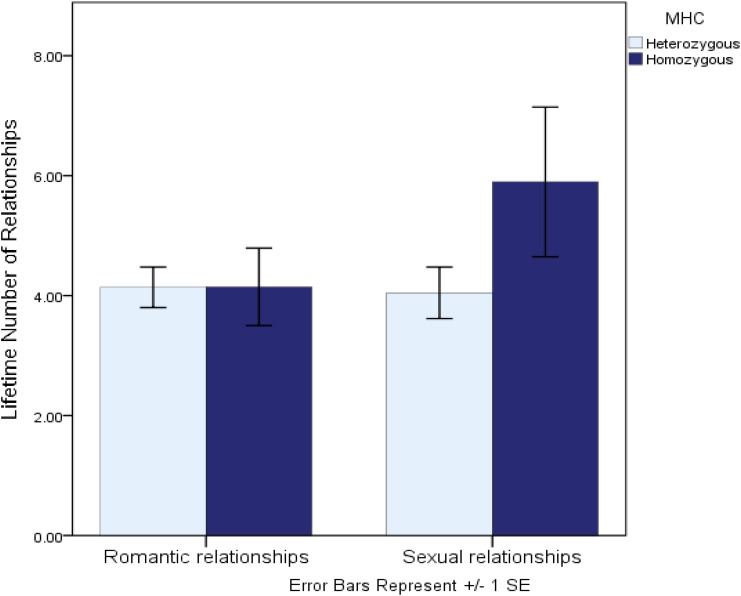
Lifetime number of romantic and sexual relationships reported by MHC-heterozygous and homozygous women (from Murray et al., 2017).

Another recent study investigated genetic variation at the MHC region of the genome—a region in which greater allelic diversity is associated with a greater ability to recognize invading non-self (vs. self) antigens. This study, conceptually underpinned by life history theory, examined whether lower heterozygosity (diversity) at the MHC—a putative marker of lower immunocompetence and thus a marker of greater future uncertainty—was associated with “faster” sexual strategies in women (Murray et al., 2017). Consistent with the predictions implied by life history theory, women who were more homozygous at the MHC region of the genome reported more favorable attitudes toward short-term mating, a more promiscuous sexual history, and perhaps most tellingly, reported being almost a full year younger when they had their sexual debut. Further comparative tests revealed that while more homozygous women reported significantly more sexual relationships, they reported an identical number of *romantic* relationships as did heterozygous women (see Figure 1). This comparative test suggests that the interpersonal implications of MHC diversity are specific to sexual strategies, rather than interpersonal dispositions more generally. Taken together, these studies suggest that genetic variants linked to chronically increased immunological vulnerabilities may also be associated with behavioral dispositions that help to either mitigate those vulnerabilities or to calibrate life history strategies accordingly.

Finally, epigenetically inspired work is beginning to investigate whether changes in disease-relevant ecological or social contexts influence disease-related gene expression. Studies from the growing area of social genomics consistently reveal transcriptional consequences of loneliness, such that people who feel lonelier (and are thus less vulnerable to socially transmitted viruses) have a relative downregulation of transcriptional elements associated with antiviral defenses and a reciprocal upregulation of pro-inflammatory transcription factors (e.g., Cole et al., 2007; Cacioppo et al., 2015). Consistent with social-cognitive perspectives on proactive defenses against potential disease threats, these transcriptional profiles are best predicted by subjective assessments (versus objective measures) of one’s environment (Cole et al., 2007; Murray et al., 2019b).

The reverse of chronic loneliness—at least physically speaking—is the formation of an intense interpersonal pair-bond. More romantically termed “love” in humans, these experiences of new love and its associated close interpersonal contact also bring with them exposure to a suite of potentially novel viruses. It is thus possible that the subjective experience of new love may be accompanied by a set of epigenetic changes designed to proactively mitigate the threat posed by exposure to new infectious threats. To date, one preliminary study supports this conceptual logic. In a multi-year longitudinal study of young women (who began the study in a new relationship, but who reported *not* yet having fallen in love), falling in love was associated with a transcriptional response consistent with a selective upregulation of antiviral defenses (characterized by upregulation of type-1 interferon response genes and a downregulation of neutrophil-related genes; Murray et al., 2019a). These transcriptional changes were independent of changes in illness, frequency of sexual activity, and self-reported loneliness. No transcriptional changes were observed in inflammation-related transcriptional profiles.

## Envoi

Although BIS and PNI research programs have largely proceeded independently of one another, the above work demonstrates that these programs of research conceptually parallel each other as well. As the lattermost social genomics work shows, logical distinctions between BIS and PNI research are quickly becoming less defensible, consistent with previous perspicacious predictions (e.g., Clark and Fessler, 2014; Gangestad and Grebe, 2014). As this type of socially inspired physiological work on the BIS expands it may, in fact, become indistinguishable from that of more classically focused psychoneuroimmunology, and their distinctions eventually pedantic.

The large rift that remains, however, must first uncover the surely numerous undiscovered pathways between multisensory perception, overt social behavior, immune function, and genes. While PNI has sought to understand how non-specific shifts in immune function impact mental health and behavior (and how non-specific stressors influence immune function), BIS-related research has focused on how pathogen cues impact behavior, as modulated by changes in physiological immunity. Researchers in each field can help to inform the research done in the other. Continued work into the physiological determinants of the BIS can complement PNI research by identifying which specific proinflammatory, anti-inflammatory, and multifunctional components of the immune system impact BIS function and vice versa in both short-term (i.e., flu season) and long-term pathogenic contexts. Further, PNI’s study of other functions of the immune system besides avoidance (e.g., defense, tolerance, maintenance) may help inspire BIS research to examine how such components might calibrate BIS function. As this research grows, distinctions between these disciplines will become ever more arbitrary.

## Author Contributions

DM, MP, and ZA all contributed to the drafting of the manuscript and making critical revisions. All authors approved the manuscript prior to submission.

## Conflict of Interest Statement

The authors declare that the research was conducted in the absence of any commercial or financial relationships that could be construed as a potential conflict of interest.
